# Supplementary material for: Reasoning action-centric temporal relations at rich feature hierarchies for action recognition
Source: PLoS One. 2025 Jul 24;20(7):e0327302. doi: 10.1371/journal.pone.0327302 (PMC12288993; doi:10.1371/journal.pone.0327302)
Supplement: S1 Appendix — (PDF) [file pone.0327302.s001.pdf]

# Jensen–Shannon Divergence

Jensen–Shannon divergence is a common indicator to statistically measure the similarity between two distributions [1]. Therefore, we leverage it to verify the observation in the main text that $dist(\bar{\mathcal{W}}_{t,t-1}, \bar{\mathcal{W}}_{t,t}, \bar{\mathcal{W}}_{t,t+1})$ is similar at different time steps. Specifically, we refer to $dist(\bar{\mathcal{W}}_{t,t-1}, \bar{\mathcal{W}}_{t,t}, \bar{\mathcal{W}}_{t,t+1})$ as $\mathbf{v}_t \in \mathbb{R}^3$ herein for simplicity, and pair-wisely calculate the Jensen–Shannon divergence for $\{\mathbf{v}_2, \mathbf{v}_3, ..., \mathbf{v}_7\}$ [2] as:

$$JS(\mathbf{v}_p, \mathbf{v}_q) = \frac{1}{2} \sum_{k=1}^{3} (\mathbf{v}_{pk} \log \frac{\mathbf{v}_{pk}}{\mathbf{v}_{pk} + \mathbf{v}_{qk}} + \mathbf{v}_{qk} \log \frac{\mathbf{v}_{qk}}{\mathbf{v}_{pk} + \mathbf{v}_{qk}}), \tag{1}$$

where $JS$ is short for the Jensen–Shannon divergence. $p$ and $q$ are both indexes in the range of $[2, 7]$.

To statistically demonstrate the results, we further calculate the mean, maximum, and minimum values of $JS$ as:

$$\begin{aligned} JS_{mean} &= \frac{1}{25} \sum_{p} \sum_{q \neq p} JS(\mathbf{v}_p, \mathbf{v}_q) \\ JS_{max} &= \max(\{JS(\mathbf{v}_p, \mathbf{v}_q)\}_{\forall p, \forall q, q \neq p}) \\ JS_{min} &= \min(\{JS(\mathbf{v}_p, \mathbf{v}_q)\}_{\forall p, \forall q, q \neq p}) \end{aligned} \tag{2}$$

where $\max(\{...\})$ and $\min(\{...\})$ respectively refer to the operators of returning the maximum and minimum values in the input set. The statistical results, as presented at the top of the main text's Fig. 4(b), all with a nearly zero value, which corroborates our observation and indicates that the graph-based reasoner tends to impose the similar local impact on different time steps.

---

[1] The value of Jensen–Shannon divergence is in the range of $[0, 1]$: The more similar two distributions are, the closer the value is to 0, and vice versa.

[2] $\mathbf{v}_1$ and $\mathbf{v}_8$ are ignored since $\bar{\mathcal{W}}_{t,t-1}$ and $\bar{\mathcal{W}}_{t,t+1}$ are respectively unacquirable in them.
