# Supplementary material for: Reasoning action-centric temporal relations at rich feature hierarchies for action recognition
Source: PLoS One. 2025 Jul 24;20(7):e0327302. doi: 10.1371/journal.pone.0327302 (PMC12288993; doi:10.1371/journal.pone.0327302)
Supplement: S2 Appendix — (PDF) [file pone.0327302.s002.pdf]

More Training Details

Herein, we provide more training details based on the demonstrations in the main text. To generate the input clips, the dense sampling strategy (sampling consecutive T frames with a fixed temporal interval τ [1]) is adopted for Kinetics dataset, and the uniform sample strategy (uniformly dividing all frames into T segments and randomly choosing one frame from each) is adopted for Something-Something datasets. The short side of each frame in a clip is fixed to be 256. Moreover, scale-jittering with the range of $[256, 340]$ and random cropping are applied as the data augmentation. And the inputs are finally resized to be 224×224 before fed into the models.

As for the training parameters, we basically follow previous works to set them [1,4,2,3]. Concretely, on Kinetics dataset, we train SlowOnly+ATR for 196 epochs, and the learning-rate is updated in the cosine style, i.e., the learning-rate at n-th epoch is $0.05[\cos(\frac{n}{196}) + 1]$. When training TSN+ATR and TSM+ATR on Kinetics, we shorten the total epochs to be 150. The learning rate is initialized as 0.1, and decreased by a factor of 0.1 at 90 and 130 epochs. A linear warm-up strategy is also applied the first 34 epochs for SlowOnly+ATR and in the first 10 epochs for TSN+ATR and TSM+ATR.

On two Something-Something datasets, all models are trained with the same policies: the learning-rate is initialized as 0.1 and decayed at 30, 40, 45 epochs

References

1. Feichtenhofer, C., Fan, H., Malik, J., He, K.: Slowfast networks for video recognition. In: Proceedings of the IEEE international conference on computer vision. pp. 6202–6211 (2019) 1
2. Jiang, B., Wang, M., Gan, W., Wu, W., Yan, J.: Stm: Spatiotemporal and motion encoding for action recognition. In: Proceedings of the IEEE International Conference on Computer Vision. pp. 2000–2009 (2019) 1
3. Li, Y., Ji, B., Shi, X., Zhang, J., Kang, B., Wang, L.: Tea: Temporal excitation and aggregation for action recognition. In: Proceedings of the IEEE/CVF Conference on Computer Vision and Pattern Recognition. pp. 909–918 (2020) 1
4. Lin, J., Gan, C., Han, S.: Tsm: Temporal shift module for efficient video understanding. In: Proceedings of the IEEE International Conference on Computer Vision. pp. 7083–7093 (2019) 1

[1] τ is fixed to be 8 in our experiments
