# Supplementary material for: Reasoning action-centric temporal relations at rich feature hierarchies for action recognition
Source: PLoS One. 2025 Jul 24;20(7):e0327302. doi: 10.1371/journal.pone.0327302 (PMC12288993; doi:10.1371/journal.pone.0327302)
Supplement: S3 Appendix — (PDF) [file pone.0327302.s003.pdf]

# More State-of-the-art Comparisons

We here extend the state-of-the-art comparisons in the main text by comparing our method with other recent-published works. The results on Something-Something V1 and V2 datasets are shown in Table 1, where our ATR also presents superior performances. In peculiar, under the 16-frames formulation, our method moves forward the state-of-the-art performances by 1.3% and 0.2% respectively on Something-Something V1 and V2.

The comparison results on the Kinetics dataset are given in Table 2. Note that our ATR focuses on enhancing the model's reasoning ability regarding how action-related objects transform over time. However, the understanding of the static scene information is critical for Kinetics. Nevertheless, we still present state-of-the-art performances on this dataset, no matter of comparison to 2D-CNN or 3D-CNN based methods.

| **Method** | **Backbone** | **Frames** | **GFLOPs ×Clips×Crops** | **Top-1 @Sth-V1** | **Top-1 @Sth-V2** |
|---|---|---|---|---|---|
| $\text{ECO}_{En}\textit{Lite}$ [16] | BNIncep+3D Res18 | 92 | 267×1×1 | 46.4 | - |
| NL I3D [12] | 3D ResNet-50 | 32 | 168×2×1 | 44.4 | - |
| TSN [6] | BNInception | 8 | 16×1×1 | 19.5 | - |
| | ResNet-50 | 8 | 33×2×3 | 20.5 | 30.4 |
| TSM [6] | ResNet-50 | 8 | 33×2×3 | 47.3 | 61.7 |
| | | 16 | 65×2×3 | - | 63.4 |
| STM [3] | ResNet-50 | 8 | 33×10×3 | 49.2 | 62.3 |
| | | 16 | 67×10×3 | 50.7 | 64.2 |
| TEA [5] | ResNet-50 | 8 | 35×10×3 | **51.7** | - |
| | | 16 | 70×10×3 | 52.3 | - |
| GSM [7] | Inception-V3 | 8 | 27×1×1 | 49.0 | - |
| | | 16 | 54×1×1 | 50.6 | - |
| MSNet [4] | ResNet-50 | 8 | 34×1×1 | 50.9 | 63.0 |
| | | 16 | 67×1×1 | 52.1 | 64.7 |
| ACTION-Net [13] | ResNet-50 | 8 | 35×10×3 | - | 62.5 |
| | | 16 | N/A×10×3 | - | 64.0 |
| MVFNet [14] | ResNet-50 | 8 | 33×2×3 | 50.5 | 63.5 |
| | | 16 | 66×2×3 | 52.6 | 65.2 |
| TSM+ATR (Ours) | ResNet-50 | 8 | 34×2×3 | 50.5 | **64.0** |
| | | 16 | 69×2×3 | **53.9** | **65.4** |

Table 1: **More state-of-the-art comparisons on Something-Something (Sth) V1 and V2.**

All the above results further confirm the effectiveness and generality of our method, and indicate the consequence of enhancing the model's temporal relational reasoning ability.

| Methods (3D-CNNs) | Backbone | Frames | GFLOPs ×Views | Top-1 |
|---|---|---|---|---|
| I3D [1] | 3D Incep V1 | 64 | 108 ×N/A | 72.1 |
| S3D-G [15] | 3D Incep V1 | 64 | 71.4×N/A | 74.7 |
| R(2+1)D [8] | 3D Res-34 | 32 | 152×10 | 74.3 |
| Nonlocal [11] | 3D Res-50 | 32 | 282×30 | 76.5 |
| SlowOnly [2] | 3D Res-50 | 8 | 42×30 | 74.9 |
| SlowFast [2] | 3D Res-50 | 32 | 65.7×30 | **77.0** |
| CorrNet [9] | 3D Res-50 | 32 | 115×10 | **77.2** |
| SlowOnly+ATR (Ours) | 3D Res-50 | 8 | 43.4×30 | 76.3 |

| Methods (2D-CNNs) | Backbone | Frames | GFLOPs ×Views | Top-1 |
|---|---|---|---|---|
| TSN [10] | ResNet-50 | 8 | 33×30 | 70.6 |
| TSM [6] | ResNet-50 | 8 | 33×30 | 74.1 |
| STM [3] | ResNet-50 | 8 | 33×30 | 73.7 |
| TEA [5] | ResNet-50 | 8 | 35×30 | 75.0 |
| MSNet [4] | ResNet-50 | 8 | 34×10 | 75.0 |
| TSM+ATR (Ours) | ResNet-50 | 8 | 34×30 | **75.7** |

Table 2: **More state-of-the-art comparisons on Kinetics.**

# References


1. Carreira, J., Zisserman, A.: Quo vadis, action recognition? a new model and the kinetics dataset. In: proceedings of the IEEE Conference on Computer Vision and Pattern Recognition. pp. 6299–6308 (2017) 2
2. Feichtenhofer, C., Fan, H., Malik, J., He, K.: Slowfast networks for video recognition. In: Proceedings of the IEEE international conference on computer vision. pp. 6202–6211 (2019) 2
3. Jiang, B., Wang, M., Gan, W., Wu, W., Yan, J.: Stm: Spatiotemporal and motion encoding for action recognition. In: Proceedings of the IEEE International Conference on Computer Vision. pp. 2000–2009 (2019) 1, 2
4. Kwon, H., Kim, M., Kwak, S., Cho, M.: Motionsqueeze: Neural motion feature learning for video understanding. In: European Conference on Computer Vision. pp. 345–362. Springer (2020) 1, 2
5. Li, Y., Ji, B., Shi, X., Zhang, J., Kang, B., Wang, L.: Tea: Temporal excitation and aggregation for action recognition. In: Proceedings of the IEEE/CVF Conference on Computer Vision and Pattern Recognition. pp. 909–918 (2020) 1, 2
6. Lin, J., Gan, C., Han, S.: Tsm: Temporal shift module for efficient video understanding. In: Proceedings of the IEEE International Conference on Computer Vision. pp. 7083–7093 (2019) 1, 2
7. Sudhakaran, S., Escalera, S., Lanz, O.: Gate-shift networks for video action recognition. In: Proceedings of the IEEE/CVF Conference on Computer Vision and Pattern Recognition. pp. 1102–1111 (2020) 1
8. Tran, D., Wang, H., Torresani, L., Ray, J., LeCun, Y., Paluri, M.: A closer look at spatiotemporal convolutions for action recognition. In: Proceedings of the IEEE conference on Computer Vision and Pattern Recognition. pp. 6450–6459 (2018) 2

9. Wang, H., Tran, D., Torresani, L., Feiszli, M.: Video modeling with correlation networks. In: Proceedings of the IEEE/CVF Conference on Computer Vision and Pattern Recognition. pp. 352–361 (2020) 2
10. Wang, L., Xiong, Y., Wang, Z., Qiao, Y., Lin, D., Tang, X., Van Gool, L.: Temporal segment networks: Towards good practices for deep action recognition. In: European conference on computer vision. pp. 20–36. Springer (2016) 2
11. Wang, X., Girshick, R., Gupta, A., He, K.: Non-local neural networks. In: Proceedings of the IEEE conference on computer vision and pattern recognition. pp. 7794–7803 (2018) 2
12. Wang, X., Gupta, A.: Videos as space-time region graphs. In: Proceedings of the European conference on computer vision (ECCV). pp. 399–417 (2018) 1
13. Wang, Z., She, Q., Smolic, A.: Action-net: Multipath excitation for action recognition. In: Proceedings of the IEEE/CVF Conference on Computer Vision and Pattern Recognition. pp. 13214–13223 (2021) 1
14. Wu, W., He, D., Lin, T., Li, F., Gan, C., Ding, E.: Mvfnet: Multi-view fusion network for efficient video recognition. In: Proceedings of the AAAI Conference on Artificial Intelligence. vol. 35, pp. 2943–2951 (2021) 1
15. Xie, S., Sun, C., Huang, J., Tu, Z., Murphy, K.: Rethinking spatiotemporal feature learning: Speed-accuracy trade-offs in video classification. In: Proceedings of the European Conference on Computer Vision (ECCV). pp. 305–321 (2018) 2
16. Zolfaghari, M., Singh, K., Brox, T.: Eco: Efficient convolutional network for online video understanding. In: Proceedings of the European conference on computer vision (ECCV). pp. 695–712 (2018) 1
