# Supplementary material for: Reasoning action-centric temporal relations at rich feature hierarchies for action recognition
Source: PLoS One. 2025 Jul 24;20(7):e0327302. doi: 10.1371/journal.pone.0327302 (PMC12288993; doi:10.1371/journal.pone.0327302)
Supplement: Attachment — Submitted filename: plos_one_response.pdf [file pone.0327302.s005.pdf]

Response to Editor's and Reviewers' Comments

May 15, 2025

We would like to reiterate our sincere gratitude to the editor and the reviewers for their insightful and supportive comments and valuable suggestions. Below, we have regrouped the sets of comments and have provided point-by-point replies. We hope that this version satisfies the request for changes necessary to proceed for publication. In order for clarity, all the answers as well as the changes in the manuscript are displayed in blue.

Response to Associate Editor

***Comment**: Overall good work, as per comments by three reviewers. However, let me share a few comments. Recently publications of top journals are expected to be discussed in related work. There is not a single paper of year 2025. How let me suggest that the proposed work should be compared with the following paper: ResLNet: deep residual LSTM network with longer input for action recognition. In addition, a few more should be discussed in related work, such as Nie, F., et al (2025). An Adaptive Solid-State Synapse with Bi-Directional Relaxation for Multimodal Recognition and Spatio-Temporal Learning. TASTA: Text-Assisted Spatial and Temporal Attention Network for Video Question Answering.*

Reply: Thank you for your constructive comments on our manuscript. We agree that incorporating recent publications is important for improving the completeness and relevance of the related work. In the revised manuscript, we have added discussions of several recent studies. These works are now included in the Related Work section (see lines 73–79, 88–90, 103–104, and 119–121). We also fully addressed all reviewers' concerns in the revised version. We sincerely hope that this revised manuscript will be considered for publication.

Response to Referee #1

***Comment 1**: The abstract provides a concise overview of the research objectives, methodology, and key findings, but incorporating a brief mention of the practical significance or deployment scenarios of the results would enhance its impact.*

Reply: Thank you for your insightful comment. In the revised manuscript, we have revised the abstract to include a brief statement on the practical relevance of our proposed method.

***Comment 2**: Introduction and Related work: The introduction is well-structured and clearly outlines the research problem while contextualizing the literature within existing studies. However, to strengthen its relevance and depth, the authors should articulate the research objectives more precisely and clearly highlight the specific research gap.*

Reply: Thank you for your insightful suggestion. In response, we have revised the introduction to more clearly articulate the research objectives and highlight the specific research gap. In particular, we emphasize that existing works relying on object detectors still suffer from background interference due to their inclusion of irrelevant objects. Additionally, we point out that these methods typically overlook low-level features and are difficult to integrate into multi-level backbones due to their heavy computational overhead and

structural inflexibility. To address these issues, we now explicitly state our goal: to formulate temporal relational reasoning in an action-centric and hierarchical manner—focusing on (1) suppressing background distractions by accurately locating action-relevant regions, and (2) enabling efficient integration with multi-level feature hierarchies. The revised paragraph can be found in the Introduction section (lines 21–33, page 2) of the updated manuscript.

***Comment 3**: The use of dense sampling for Kinetics and uniform sampling for Something-Something is appropriate, but adding a one-line justification for each (e.g., balancing efficiency vs. temporal coverage) would improve clarity. Additionally, the phrase "3-crops of 256 × 256" could be clarified does cropping occur after resizing the short side to 256?*
Reply: Thank you for your suggestion. We have addressed your comment by adding one-line justifications for the sampling strategies used for Kinetics and Something-Something datasets. Specifically, we clarify that dense sampling is adopted for Kinetics due to its relatively static actions, where capturing discriminative spatial details is crucial. In contrast, uniform sampling is used for Something-Something, which contains temporally structured actions, to ensure broader temporal coverage while maintaining computational efficiency (lines 269-276, page 8). In addition, we have revised the phrase "3-crops of 256×256" for clarity. We now explicitly state that the cropping is performed after resizing the shorter side of the frame to 256 pixels (lines 280-283, page 8).

***Comment 4**: The ablation study section is systematically presented; however, the authors should revise and expand the interpretations to provide more detailed and insightful analysis.*
Reply: Thank you for your suggestion. Following your suggestion, we have carefully revised and expanded our interpretations to provide more detailed and insightful analysis. The revised explanations can be found in lines 298–317, lines 321-326, and lines 332-349.

***Comment 5**: The author should strongly revise and extend the conclusions section.*
Reply: Thank you for your suggestion. In response, we have thoroughly revised and extended the conclusion to better highlight the contributions and findings of our work. Please see the lines 426-437 on page 13.

Response to Referee #2

***Comment 1**: Comparison with newer models: It would strengthen the paper to benchmark against recent transformer-based methods (e.g., TimesFormer, VideoMAE)*
Reply: Thank you for the suggestion. We have added the performance of TimesFormer in Table 6 (page 12). For VideoMAE, we do not include a direct comparison, because it is a video masked autoencoder that employs a self-supervised learning approach to obtain more effective video representations during pre-training. In contrast, our proposed method adopts a supervised learning strategy.

***Comment 2**: Efficiency Reporting: In addition to FLOPs, providing inference time and parameter size would give a fuller view of the model's efficiency.*
Reply: Thank you for the valuable suggestion. In addition to reporting FLOPs, we have now included the parameter size of our model in the revised manuscript (see Table 6 on page 12).

***Comment 3**: Limitations Discussion: A brief mention of potential limitations (e.g., transferability to highly complex scenes or multi-action scenarios) would add balance.*
Reply: Thank you for the suggestion. We have added a discussion of the limitations in the revised version (lines 380–387, page 12).

***Comment 4**: Kinetics Analysis: Since Kinetics is less motion-focused, a more detailed discussion on ATR's improvements there would be valuable.*
Reply: Thank you for the suggestion. As suggested by the reviewer, we have added more discussions on ATR's improvements. (lines 359–366, page 11)

Comment 5: *Line 5: "academic and industrial communities." Consider adding a reference supporting this claim.*
Reply: We sincerely appreciate the valuable comments. We have added two references after "academic and industrial communities".
1. Wang M, Xing J, Jiang B, et al. A multimodal, multi-task adapting framework for video action recognition[C]. AAAI. 2024, 38(6): 5517-5525.
2. Chen J, Ho C M. MM-ViT: Multi-modal video transformer for compressed video action recognition[C]. WCACV. 2022: 1910-1921.

Comment 6: *Fig. 2 caption: "Design details of ARL are demonstrated in Fig. 3" maybe say "explained" instead of "demonstrated" for better English flow.*
Reply: Thank you for the helpful suggestion. We have revised the caption of Fig. 2 by replacing "demonstrated" with "explained" to improve the clarity and fluency of the expression.

Comment 7: *Page formatting: Be cautious of occasional broken lines across pages (e.g., equations (1), (2), etc.).*
Reply: Thank you for the suggestion. We have addressed this in the revised manuscript.

Comment 8: *References: The style is slightly inconsistent in some parts (e.g., mixing arXiv preprints with conference papers); better formatting may be needed for final publication.*
Reply: Thank you for the suggestion. We have checked the references carefully and corrected the errors accordingly in the revised manuscript.

Response to Referee #3

Comment 1: *The writing needs better grammar and clarity in several places, with some phrases being unclear.*
Reply: Thank you for your suggestion. We have carefully polished the language in the revised manuscript to enhance clarity and readability.

Comment 2: *The paper does not compare its method to transformer-based video models, which are important recent baselines.*
Reply: Thank you for the suggestion. We have added the performance of TimesFormer in Table 6 (page 12).

Comment 3: *Some key settings (like window size and number of layers) are fixed without testing how much they affect the results.*
Reply: Thank you for the suggestion. Table 1 presents the results with different numbers of ATR blocks. We observe a consistent performance improvement as more ATR blocks are inserted. For the window size k, as discussed in the Visualization Analysis (line 197, page 6), the most activated elements in each row are typically centered around the current time step and its two adjacent temporal neighbors. Additionally, the low Jensen–Shannon divergence values suggest that the reasoner applies a relatively consistent local influence across time steps. Thus, increasing the window size k does not lead to further performance gains.

Comment 4: *The paper does not mention any possible limitations, such as how the method performs on long or complex videos.*
Reply: Thank you for your valuable comment regarding the discussion of potential limitations. In the revised manuscript, we have added a specific paragraph (lines 417–424, page 13) to explicitly address the limitations of our proposed method. While our approach achieves strong performance across multiple backbones and datasets, we acknowledge two main limitations: 1) Transferability to complex or multi-action scenarios. 2) Lack of spatial relational reasoning in the Efficient Reasoner.
