# Supplementary material for: Reasoning action-centric temporal relations at rich feature hierarchies for action recognition
Source: PLoS One. 2025 Jul 24;20(7):e0327302. doi: 10.1371/journal.pone.0327302 (PMC12288993; doi:10.1371/journal.pone.0327302)
Supplement: Attachment — Submitted filename: Recommendation.docx [file pone.0327302.s004.docx]

**Author Comment**

The authors propose a novel Action-centric Temporal-relational Reasoning (ATR) block for enhancing action recognition by focusing on action-centric and hierarchical temporal relation reasoning. The work is well-organized, the methodology is clear, and the experimental results convincingly demonstrate the proposed method's effectiveness across multiple datasets and baseline architectures. Visualizations further support the claims. Overall, this work addresses a critical limitation in current methods (background distraction and lack of multi-level reasoning) and offers a promising solution.

**Strengths:**

- Innovative formulation of action-centric and hierarchical temporal reasoning.
- Strong experimental validation across multiple datasets (Something-Something V1/V2, Kinetics).
- Clear methodology with detailed ablation studies and insightful visualizations.
- Lightweight and flexible design, easily pluggable into existing architectures.

**Weaknesses and Suggestions:**

- **Comparison with newer models:** It would strengthen the paper to benchmark against recent transformer-based methods (e.g., TimesFormer, VideoMAE).
- **Efficiency Reporting:** In addition to FLOPs, providing inference time and parameter size would give a fuller view of the model’s efficiency.
- **Limitations Discussion:** A brief mention of potential limitations (e.g., transferability to highly complex scenes or multi-action scenarios) would add balance.
- **Kinetics Analysis:** Since Kinetics is less motion-focused, a more detailed discussion on ATR's improvements there would be valuable.

**Minor Points (Typos/Formatting):**

- Line 5: "academic and industrial communities." Consider adding a reference supporting this claim.
- Fig. 2 caption: "Design details of ARL are demonstrated in Fig. 3" maybe say "explained" instead of "demonstrated" for better English flow.
- Page formatting: Be cautious of occasional broken lines across pages (e.g., equations (1), (2), etc.).
- References: The style is slightly inconsistent in some parts (e.g., mixing arXiv preprints with conference papers); better formatting may be needed for final publication.

**N. B.:** Very good work; addressing the above points will further enhance the paper's quality.
